# An emergentist *vs* a linear approach to social change processes: a gender look in contemporary India between modernity and Hindu tradition

**DOI:** 10.1186/s40064-015-0933-7

**Published:** 2015-04-01

**Authors:** Rosalia Condorelli

**Affiliations:** Department of Political and Social Sciences, Catania University, 8 Vittorio Emanuele II, Catania, 95131 Italy

**Keywords:** Gender inequality, Indian patriarchy and Hindu tradition, Dowry practices, Indian widowhood, Mutual reinforcement between tradition and modernity, Complex adaptive social systems, Emergentist *vs* linear social change process

## Abstract

Using Census of India data from 1901 to 2011 and national and international reports on women’s condition in India, beginning with *sex ratio* trends according to regional distribution up to female infanticides and sex-selective abortions and dowry deaths, this study examines the sociological aspects of the gender imbalance in modern contemporary India. Gender inequality persistence in India proves that new values and structures do not necessarily lead to the disappearance of older forms, but they can co-exist with *mutual* adaptations and reinforcements. Data analysis suggests that these unexpected combinations are not comprehensible in light of a linear concept of social change which is founded, in turn, on a concept of social systems as linear interaction systems that relate to environmental perturbations according to proportional cause and effect relationships. From this perspective, in fact, behavioral attitudes and interaction relationships should be less and less proportionally regulated by traditional values and practices as exposure to modernizing influences increases. And progressive decreases should be found in rates of social indicators of gender inequality like dowry deaths (the inverse should be found in *sex ratio* trends). However, data does not confirm these trends. This finding leads to emphasize a new theoretical and methodological approach toward social systems study, namely the conception of social systems as *complex adaptive systems* and the consequential e*mergentist, nonlinear* conception of social change processes. Within the framework of e*mergentist theory* of social change is it possible to understand the lasting strength of the patriarchal tradition and its problematic consequences in the modern contemporary India.

**Electronic supplementary material:**

The online version of this article (doi:10.1186/s40064-015-0933-7) contains supplementary material, which is available to authorized users.

## Introduction

This study examines the sociological aspects of the gender imbalance in contemporary India. Although contemporary India presents itself on the world scene as a country with some traits of undoubted modernity, above all considered in its dimension of cumulative process of technological innovation and economic development and formal juridical parity between sexes, Indian patriarchy and Hindu tradition with its less individualistic contents, in particular those contents mortifying the identity of women establishing their subordination to male power, continues to persist and resist attempts of redefinition and of internal renewal. Between tradition and modernity, gender inequality persistence in India proves that new values and structures do not necessarily lead to the disappearance of older forms, which continue to last even as an emergent effect of unexpected combinations of tradition and modernity. This is already an acquired finding in current Sociological literature.

The sociological analysis of the dynamics of social change in today’s complex societies restricts the spectrum of reference to be assigned to a linear conception of social change, assuming a proportional contraction of tradition’s action sphere as modernization progresses. It is now accepted, that modern institutions and values do not simply replace traditional ones. The relations between modern and traditional forms do not necessarily involve a polar opposition, a mutual exclusion or conflict, and therefore do not necessarily lead to social dichotomization (modern societies *vs* traditional societies). But manifold combinations and also surprising *emerging* scenarios constitute a wide range of possible alternatives (non unilinear models of social change and *multiple forms* of tradition and modernity was proposed from Gerschenkron 1962, Gusfield 1967, Eisenstadt 1973). Traditions may continue to persist and influence the acceptance of modern elements, their *fusion* and *reciprocal reinforcement*. So, modernity, harnessed to more traditional orientations, supports tradition and *vice versa.* The analysis of these possible social change dynamics finds a vast spectrum of reference in present modern complex societies.

From this standpoint, as I said, India presents itself on the world scene as a country with some traits of undoubted modernity. In his study on the dialectical relationship between religion and society, Hindu cultural and religious tradition appeared to Weber as the main cause of social immobility and impediment to modernization, to the progress towards those forms of individualism, acquisitiveness, and economic rationalization which innervate modern capitalistic societies. Today’s Indian society does not confirm, indeed, this polar opposition between tradition and modernity. It presents a more complex scenario. Modernity has made its way and has been accepted, combined with and filtrated through tradition, which has acted to absorb the modern, adapting it to itself and even using it to its own advantage, ensuring its persistence. New changes have not, therefore, displaced older traditional orientations, but they show that they can co-exist with *mutual* adaptations.

In the past, Gusfield (1967) had found abundant proof of this process in India. He observed, for example, that, after English Colonialism, the attempt to construct a modern united Indian nation relied on a not so modern element such as Hindu nationalism in order to gain consensus and legitimacy of the political authority. It was also undeniable, according to Gusfield, that a considerable contribution to the modernization process was due to the functional specialization attached to the caste system. It was likewise evident that modernization with its individualistic criteria of free vertical mobility had contributed to Brahmanism. The ‘modern’ criteria of social mobility seemed to have been accepted, re-modulated and harnessed to more traditional orientations by the middle classes, as evidenced by its reformulation in the equivalent of a inter-caste vertical mobility process (*sanskritization process,* see note 3)*.* These observations are still extraordinarily relevant today, considering present day Indian political events which have brought to power the BJP Hindu party.

Even the matter of gender discrimination confirms this process and will be the subject of this article. In spite of formal equality between the sexes, Indian patriarchy and Hindu tradition not only continues to persist, but its persistence is also due to *mutual* adaptations between tradition and modernity. Modernization, considered in its materialist and technological innovation dimensions, has been accepted from traditional value profiles and has even reinforced the sexist practices of tradition. For instance, the success of modern sex-selective abortion techniques can only be explained by taking into account the sexist nature of Hindu tradition. Sex-selective abortion techniques have now become the modern surrogate of the practice of female infanticide. In the same manner, the materialism of modern consumerism has been accepted and incorporated into traditional mechanisms of the dowry system, which has found in modernity support and further reason for its social desirability. Modernization has reinforced its persistence and encouraged, despite increased participation of women in the market economy, its ever wider spread throughout the territory and social classes/castes^a^, along with all its well known negative implications such as dowry deaths and female infanticides and feticides. Quoting Shils’ study on Indian intellectuals between tradition and modernity (Shils 1961), Gusfield (1967) remarked how even highly Westernized Indian intellectuals and urbanized elites were deeply tied to Hinduism and Indian family life. Still today, strong ties with Hindu tradition and extended family, compliance with parental arrangement as dominant system of marital selection and a traditional conception of marital roles are found among westernized and urbanized elites as well as in the provincial and rural villages. With a chain reaction, the ‘modern’ criteria of vertical social mobility, accepted and reformulated within Hindu tradition by the low and middle castes/classes, has proved to be functional both for the maintenance of Brahmanism and indirectly for the conservation and enhancement of the dowry system and consequentially of female infanticide and feticide practice in the low and middle caste.

The above being stated, this study examines the sociological aspects of the gender imbalance in contemporary India, showing how old traditions that justify discrimination against women and widows persist in a society where the Indian Silicon Valley, digital technology, modern university Campuses, the election of a woman President, Miss World Competitions and Bollywood coexist along with the signs of *unprecedented* discrimination against women and widows (Giri 2002a, p. 107), dowry practices and dowry deaths, female infanticides and feticides, the holy city of Vrindavan and its ashrams. Our interest is geared toward *unexpected, emergent*, combinations between modern forms and older traditional gender forms existing in contemporary Indian society. From our perspective, these unexpected combinations should be framed within a new theoretical and methodological approach toward social system study, namely within the conception of social systems as *complex adaptive systems* and an e*mergentist, nonlinear* conception of social change. This aspect will be addressed in the concluding section of the article. Data supporting our point of view, which represent the major social indicators of gender inequality, beginning with *sex ratio* trends according to regional distribution up to female infanticides and sex-selective abortions and dowry deaths, come from official census data from 1901 to 2011 and from national and international reports on women’s condition in India.

### Religion and society: the interpretive context of women’s marginalization in India

A complex interweaving of relations deriving from patriarchal structures, types of economic structures, upon which depend women’s involvement in production activities and consequently the degree of their autonomy and social value, and Hinduism explain power imbalance between the sexes and the persistence of a strong state of female subordination and marginalization in contemporary Indian society.

On the one hand, it is quite true that the Indian Republic recognized equal rights for all citizens after Independence, regardless of sex, class and caste. Laws were enacted which, contrary to patriarchal and religious traditions, guaranteed women the right to inheritance, to divorce, to a second marriage in case of widowhood (forbidden by the *Manu Code*), the right to education and to free and equal occupational retribution, and the right to vote. Moreover, Laws prohibit customs such as *sati* (the practice of immolating widows on their husbands’ funeral pyre), child marriages, polygamy, the dowry and severely punish any form of domestic violence and violence concerning dowries. Nevertheless, to this formal equality corresponds a *de facto* inequality.

The patriarchy social structure, banned customs, Hindu religious precepts establishing male superiority over woman continues to last. So, *de facto*, women still have a severely inferior social status compared to men. The *sex ratio* at birth, the child sex ratio between 0 to 6 years, the sex ratio from 7 years of age and over, rates of female infanticide, sex-selected abortions, literacy rates, rates of female malnutrition and vaccinations are all indicators the consistency of which witness a decisive disadvantage of women compared to men.

Sociological studies have shone that the way gender and gender boundaries are constructed in time and space, determining women’s status in social hierarchy and influencing wide spheres of social action in private and public life, is closely connected to the incidence of two principal variables: the degree of female participation in economic activity and, therefore, their economic contribution, and the degree of control women exert on economic resources. If the social and political importance of women has grown in contemporary Western societies, it is also due to the increase of the number of women who have entered the labor market. Likewise, gender studies regarding India have shone the fundamental role of these variables in explaining the women’s social value and status and, consequently, gender inequality, with a strong discriminatory power between India and other world regions (West Africa, for example, in Boserup’s classic work; Boserup 1970) as well as among Southern and Northern regions of India, among Northern and Southern rural areas, and among rural and urban areas.

In spite of the modernization process activated after colonialism which today makes India a sort of economic miracle, most of the mainland is still rural. In fact, despite the growth of a vast gamma of modern industries such as software and a noteworthy development in the service sector, about half of the labor force, out of one billion and 211 million inhabitants, is employed in agriculture and *circa* 70%, the vast majority of the population, lives in rural areas, in hinterland zones where ties to Hindu and Patriarchy traditions are more strong than urban areas (Census of India, 2011). Soon after India’s Independence, capitalistic development and economic liberalism, *laissez-faire policy* brought about an unequal distribution of wealth among geographical regions, rural and urban areas and social groups, favoring only a small part of the population (only 10% possesses 33% of the nation’s wealth, while a quarter of the population is at the poverty threshold - $0.40 *per diem*, thereby creating noteworthy social internal unbalance (Additional file 1: Table A, presenting Indian population distribution separated into wealth quintiles by urban–rural locations and by state, shows as mainly rural population concentrates in the poorest quintile compared to urban population being in richest quintile; *NFHS-3* 2005*–*2006). First, in this predominantly rural environment, women’s social condition is affected from farming system.

In the North, control of economic resources by women is non-existent and the influence of Patriarchy is persistent and pervasive. The Patriarchate institution shows a close link to the structure of production processes, to the way this structure determines the degree of female participation in production and the consequential social respect of women. In Northern rural villages like Punjab, Haryana and Uttar Pradesh, the intensive wheat cultivation and general farming system is entrusted entirely to men *(male farming system*) as it requires the use of heavy machinery such as oxen-led plows. Women, unable to handle such heavy equipment needed for that type of farming, are excluded from the agricultural production process or are allowed to participate only occasionally for short periods of time, such as during harvest time. Such an exclusion from the economic system implies social exclusion as well. By not working, women are unable to contribute in any way to the wealth and support of their family and of the community. They do not have their own income, property and are mainly involved in domestic work. Without any education and self sustenance, they are subject to the decisions, choices and often to the mood swings of their respective husbands. Therefore, they are *socially invisible,* and a *social burden,* more than a value for society. This explains the greater use of such strategies as for example, female infanticides, arranged child marriages, traditional dowry practices and dowry deaths (frequent uxorcides by husbands eager to remarry, once their wife’s dowry is consumed) and afterwards, at the husband’s death, the traditional abandonment of widows in Vrindavan or Varanasi.

The South has a denser concentration of urban and industrialized areas and more agricultural areas where the types and systems of production allow women greater participation to production processes, denied them in the North. Higher women participation in the production process in the South, due to rice cultivation (i.e. Andhra Pradesh, Tamil Nadu, Karnataka), explains why women’s status in the South is higher compared to women from Northern India, although this is not enough to guarantee women autonomous resource management and complete autonomy from male power. Traditional patriarchal and religious constraints, although more *malleable* and flexible and not as limiting as elsewhere, are present and very active. Even here in the South, there are signs of a process that sees women, from their birth, in a very vulnerable condition due to lasting dowry practices and *sanskritization process*^*b*^*.*

If today, we see educated women who work and occupy even high professional and political posts in India, this is however, only a small minority, belonging mostly to the high and middle classes who reside in the more economically advanced areas, in the South, like Kerala (Additional file 1: Table A) and more industrialized and urbanized Pondicherry (they have for example a *sex ratio* favoring women, respectively 1084 and 1038 women per 1000 men in 2011, and higher female literacy rates - with a value of 4.04% gender gap being the lowest in the country and lower than Northern regions such as Kashmir 29.25%, 18.61% of Haryana, 19.98% of Uttar Pradesh, 27.85% of Rajasthan, and again 10.14% of Punjab and 11.51% of Bengala, *Census of India* 2011). In the complex world of the Indian work force just a few women are employed in large commercial enterprises, holding top jobs of prestige and high salary (only 5% from 2009–2012, according to the *Gallop Poll*). The majority of women are for the most part employed in agricultural activities, domestic housework and occasional employment. Despite the fact that laws have been enacted to encourage and to protect women’s interests, women still continue to be victims of exploitation and marginalization. In public sectors, for example, which hire the greatest number of women, despite equal pay laws (*The Equal Remuneration Act* 1976) women still receive lower wages and salaries compared to their male co-workers.

On the whole, according to the *Global Gender Gap Reporter* 2012, published *by the World Economic Forum* which monitors the extent of gender inequality on the basis of criteria relating to access to financial resources, education and health, India ranks 105^th^ out of 135 nations considered.

Although gender imbalance is rooted in history, in economic factors, cultural traditions of a rigidly patriarchal society, with a extended patrilinear (Agnatic kinship) and patrilocal family structure, the *Manu Code* (5^th^ Century BC), indisputable source of Hindu law and morals^c^, *religiously* justifies male superiority over the female, equaling the female *virtue* to the identity of mother and faithful, obedient wife, remaining chaste and self-restrained after husband’s death until her death.

Classical Sociology theory has extensively outlined a complex interweaving of relationships between religion (*superstructure*) and society (*structure*). Religion can be influenced by society dynamics and, in turn, can influence these dynamics according to a mutual conditioning relationship. On the hand Religion can be a source of social conflict and many religious movements have favoured a change in existing social systems, on the other hand it remains an instrument of social integration and, at the same time, can work to legitimize existing power rapports, intervening to justify norms and social institutions on the basis of beliefs and representations that give to consolidated social relationships a sacred valence. Legitimating religiously social order, it can contribute to justify prevailing ways of political organization and economic organization of which it can be a reflection. Likewise, in case of Hinduism, the link between structure and superstructure appears to be very strong and circular. On one hand, economic structure influences the social set up and existing power relations, creating inevitable disparity and caste and gender inequality. On the other hand, religious Hindu “superstructure”, which may or may not be a simple epiphenomenon of the structure^d^, appears to strengthen and legitimize the set up of power relationships and social inequality that are defined by the structure itself. Hinduism, still rooted in Indian society as it was thousands of years ago, *religiously* legitimizes the structure of patriarchal social relations determined by the economic set-up and consolidates, with its chain of births and deaths (*samsara*), governed by *karman* retributive laws, the existing social, caste and gender, order. The Hinduism’s structure come moreover from changes of the original Vedic principles of equality among the sexes due to changes and disruptions of the economic and political structures of Indian society starting from 500 B.C., which confirms the link between structure and superstructure^e^.

Hinduism or, as Hindu worshippers call it, *Vaidikadharmam* inspires all of Indian culture It constitutes an entire vision of the world, which strictly establishes the way of being, behaving and understanding of both individual and social life. A set of very ancient principles and rules governs the Hindu community and promotes a holistic conception of social existence where the individual and his needs or rights are subordinate to those of the community and what the community establishes to be *the good* for the individual and the community itself. The traditional Hindu idea of compensation and metempsychosis works as an effective mechanism of social control, ensuring obedience to *collective Telos,* to duties set by caste affiliation and gender. The union between *Dharma, Karman and Metempsychosis* obliges everyone to obey, as this is necessary in order to have “a new and better “rebirth in that cycle of purification which leads to the supreme spirit, the *Brahman*. Only action (*karman),* that is, the striving to live in accordance with the rights and *duties* imposed by casteism (*Dharma*), ensures liberation (*moksa*) from the life and death cycle (*samsara*), purifies and elevates each one to Nirvana, to the ultimate beatitude with the extinction of Karman. Thus, Karman is a reward and remuneration; *Karmic* law is a law of compensation, a law of cause and effect which accompanies the concept of reincarnation. In fact, in the endless cycle of births, deaths and rebirths, the type of rebirth and therefore the possibility of improving caste collocation in a successive life depends entirely upon the merits or demerits earned in previous existences. Altough the ideas of metempsychosis and of compensation have a close connection to a deep sense of proportionality ethics (Bendix 1977), nevertheless, the idea of compensation and of caste *sacralization* legitimizes a rigidly hierarchical, socially stratified order. Hinduism operates as an instrument of social control, intervening to stabilize the set up of power relationships and a strong caste and gender discrimination.

As we said, the *Manu Code* traditionally codifies female inferiority and gender inequality. Here, caste theory set women to the fourth caste of the *Shudra* (servants) or outcasts along with the *paria.* In fact, the image of women is twofold. On the one hand they are valued in their role as mother (Doniger and Smith 1991, *The Law of Manu*, ch. 9, 96), whereas on the other hand they are *the* symbol of sexual disorder, whose explosive potentiality has to be restrained and regulated to avoid disruptive effects on social order. So, segregation and male dependence of women is justified from Hinduism at a *natural* level. The strong mark of a Biological Innatism of Theological root underlies Hinduism, which *naturalizes* and universalizes gender differences by embedding them in a creation act for which the qualities of femininity consist in *essential* traits of sensuality, fickleness, unfaithfulness, and, consequently, unability to self manage, to pursue a proper self-direction (*The Law of Manu,* ch. 9, 14–16). Therefore, for Induism the divine will is clear in depriving women of autonomy and independence, by placing them under the exclusive authority and strenuous guarding of their fathers, their husbands, or their sons in case the husband dies (*The Law of Manu,* ch, 9, 2–3; ch. 9, 14–16; Appendix). Impossibility to change a divine rule justifies, religiously, the male superiority over women in social order. This superiority implies a predominance of male roles in the public sphere whereas the *scriptural prison* relegates women in the home and makes the family the main place of their self-realization. Women’s only duty, their only *Dharma,* is being mothers (preferably to male offspring) and *virtuous* wives, in accordance with a virtue concept involving duties of obedience, faithfulness, unconditional worship to husbands even if they are devoid of “good qualities”, self-restraint and complete chastity after their death, in order to acquire Karmic merits (*The Law of Manu,* ch. 5, 154; 156; 158; 160; 161; Appendix). Dharma or total dedication to their spouse (*patrivatrya*), in life and even after husband’s death, and the punishment to endless reincarnations and rebirths as abject creatures (impure animals or outcasts) in the case of disobedience (*The Laws of Manu*, Chapter 5, verses 164, 165; Appendix), condemns women to social submission under male power. In this sense, Hinduism is a *stronghold* of patriarchy. It continues to consolidate power imbalance between the genders springing from the production setup and patriarchal tradition. On one hand, the degree of women’s participation in production which determines her *social value* and the resulting society’s patriarchal structure favor their state of inferiority. On the other hand, Hinduism, by linking women’s virtue or *moral value* to the carrying out of spousal duties involving various forms of male dependence, religiously legitimates this inferiority and enshrines *de facto* her submission to male dominance.

In conclusion, Hinduism codified and produced a conception of femininity which is still everywhere in the Indian subcontinent as a cultural heredity, ensuring the persistency of traditional authority principle on modern rationality principle. Beyond minor or major orthodoxy according to local traditions and despite Gandhi reforms ^f^ and feminist movements of the 80’s as well as current NGOs (Giri 2005), today, gender discrimination within the family and society touches women’s lives in various ways. And above all, Hindu religion thereby ends up legitimizing forms of serious *social de-responsiblization* in regard to women and in particular to widows. As sociological studies on the relationship between individual and community outlined, this is a perverse effect of solidarity founded on the principle of the primacy of community over individual (i.e. Sen 1999). The Brahminical concept of ideal wife, totally devoted to husbands, subjected to their control for her *natural* moral imperfections, renders actions of severe female marginalization devoid of any sense of *social guilt* and *responsibility*, making female subordination ‘normal’ to all concerned (including perhaps to female victims themselves, i.e. see Giri 2002b, p 11).

### Missing women: Unexpected combinations between Hindu tradition and modernity

**Figure 1:**
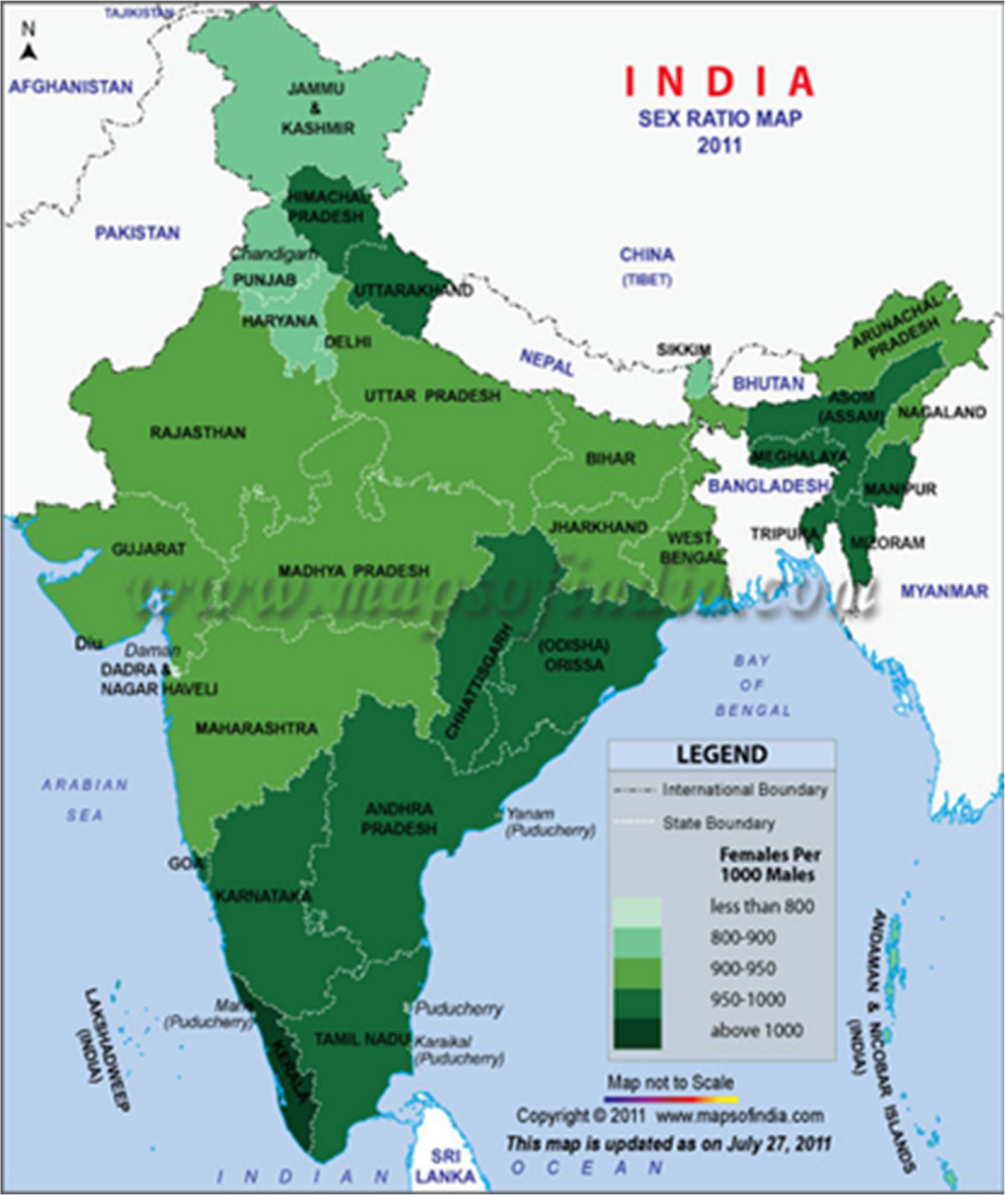
**Sex ratio of total population in India (2011).** ***Source: Census of India 2011.***

Social and Religious Institutions explain the state of female subordination and marginalization in India. As we said, the patriarchal and Hindu conception, electively assigning women the role of mother and wife and justifying their subordination to male power, persists and results in *old* and *new* forms of discrimination, whose persistence is also the result of unexpected combinations between tradition and modernity. This process of mutual reinforcement can be observed regarding to one of the oldest gender discrimination practices in India: babies girls’ suppression practice at birth. This practice is still widespread in the whole country and involves all social classes/castes due to reasons attached to the degree to women partecipation in the productive system (the more women are an economic burden and become culturally and socially devalued, the more their condition degenerates as far as to include *infanticides* and *feticides*), the diffusion of *sanskritization process* in the low and middle castes, and the haltering system of dowries. Female infanticide and feticide persistence, above all, contributes in explaining why an unfavorable female *overall sex-ratio* can be found in almost every State in India (Table 1; Figure 1):

**Table 1:** **Overall sex ratio in India 1901 – 2011 per State**

	**1901**	**1911**	**1921**	**1931**	**1941**	**1951**	**1961**	**1971**	**1981**	**1991**	**2001**	**2011**
**INDIA**	**972**	**964**	**955**	**950**	**945**	**946**	**941**	**930**	**934**	**927**	**933**	**940**
Andamane & N.	318	352	303	495	574	625	617	617	760	818	846	878
Andhra Pradesh	985	992	993	987	980	986	981	981	975	972	978	992
Arunachal P.	ND	ND	ND	ND	ND	ND	894	894	862	859	901	920
Assam	919	915	896	874	875	868	869	869	910	923	932	954
Bengala	945	925	905	890	852	865	878	878	911	917	934	947
Bihar	1061	1051	1020	995	1002	1000	1005	1005	948	907	921	916
Chandigarh	771	720	743	715	763	781	652	652	769	720	773	818
Chhatisgarh	1046	1039	1041	1043	1032	1024	1008	1008	996	985	990	991
Dadra Nagar	960	967	940	911	925	946	963	963	974	952	811	775
Daman & Diu	995	1040	1143	1088	1080	1125	1169	1169	1062	969	709	618
Delhi	862	793	733	722	715	68	785	785	808	827	821	866
Goa	1091	1108	1120	1088	1084	1128	1066	1066	975	967	960	968
Gujarat	954	946	944	945	941	952	940	940	942	934	921	918
Haryana	867	835	844	844	869	871	868	868	870	865	861	877
Himachal P.	884	889	890	897	890	912	938	938	973	976	970	974
Jammu&Kasmir	882	876	865	865	869	873	878	878	892	896	900	883
Jharkhand	1032	1021	1002	989	978	961	960	960	940	922	941	947
Karnataka	983	981	969	965	960	966	959	959	963	960	964	968
Kerala	1004	1008	1011	1022	1027	1028	1022	1022	1032	1036	1058	1084
Lakshadweep	1063	987	1027	994	1018	1043	1020	1020	975	943	947	946
Madhya P.	972	967	949	947	946	945	932	932	921	912	920	930
Maharastra	978	966	950	947	949	941	936	936	937	934	922	925
Manipur	1037	1029	1041	1065	1055	1036	1015	1015	971	958	978	987
Meghalaya	1036	1013	1000	971	966	949	937	937	954	955	975	986
Mizoram	1113	1120	1109	1102	1069	1041	1009	1009	919	921	938	975
Nagaland	973	993	992	997	1021	999	933	933	963	886	909	931
Orissa	1037	1056	1086	1067	1053	1022	1001	1001	981	971	972	978
Pondicherry	ND	1058	1053	ND	ND	1030	1013	1013	985	979	1001	1038
Punjab	832	780	799	815	836	844	854	854	879	882	874	893
Rajastan	905	908	896	907	906	921	908	908	919	910	922	926
Sikkim	916	951	970	967	920	907	904	904	835	878	875	889
Tamil Nadu	1044	1042	1029	1027	1012	1007	992	992	977	974	986	995
Tripura	874	885	885	885	886	904	932	932	946	945	950	961
Uttar Pradesh	938	916	908	903	907	908	907	907	882	876	898	908
Uttaranchal	918	907	916	913	907	940	947	947	936	936	964	963

*Women per 1000 men.*

***Source:*** *Census of India 2011.*

**Figure 2:**
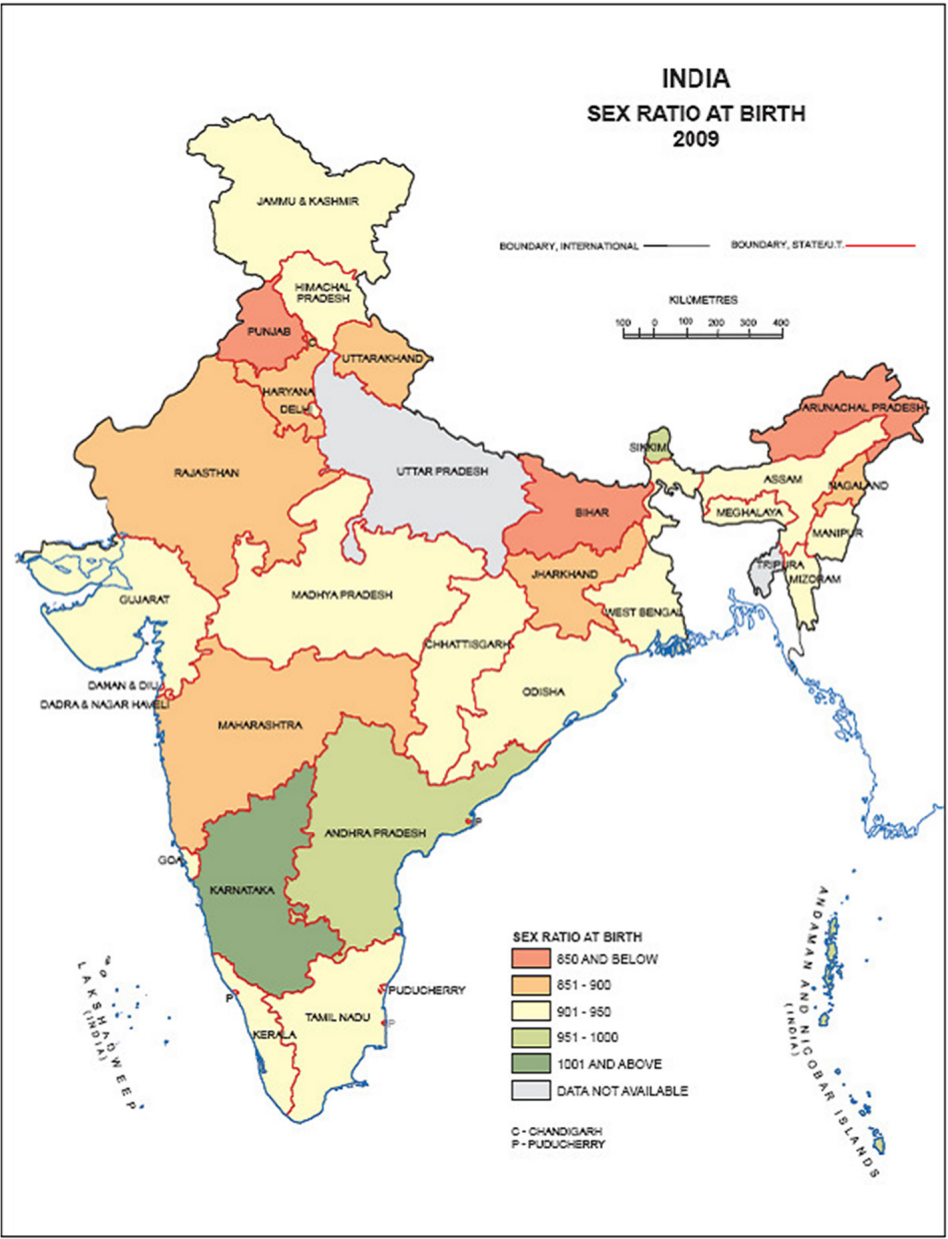
**Sex ratio at birth by state. Source: Annual report on vital statistics of India based on CRS-2009.**

On the whole, from the Census 1901 to Census 2011, the *overall sex ratio* reveals a lower presence of women within the total population, with a progressive decreasing trend (for example, from 1901 to 2001 the *overall sex ratio* declines from 972 women per 1000 men to 933, and goes to 940 in 2011, with a slight increase which, however, does not change the unfavorable general picture. The significance of this slight increase is affected by decrease of the *sex ratio at birth* - *SBR,* number of female babies born per 1000 males born - and *child sex ratio* 0*–*6 years - *CSR*, girls per 1000 boys under age 7. This applies to the 7 and over *sex ratio* trend as well; Tables 2, 3 and 4)^g^. It is no coincidence that the tendency of a lower female population out of the total population is mainly found in the rural States of Northwest compared to Indian Southern States (for example, Uttar Pradesh, Punjab, Haryana, Bihar, Rajasthan). Similarly, the *sex ratio at birth*, affecting *CSR,* is unfavorable to females and presents a similar geographical distribution as the one observed for the *overall sex ratio* (Tables 3 and 1; Figure 2). Several studies confirm that regional variability of women/men ratio shows a certain connection between the type of agriculture and female mortality: the pre and post natality female mortality increases where women do not produce wealth with the consequential rigid persistence of the patriarchal, patri-local and patri-linear, and Hindu cultural traditions, *independent* from local economic development (two of rural Northwest India’s wealthiest states (population is in the highest wealth quintile; see Additional file 1: Table A) present the lowest *sex ratio at birth* and *child sex ratio* 0–6 years: the rural states of Punjab, that has benefitted from the Agrarian Reform and the Green Revolution and Haryana, which was once part of Punjab; the same is true for Gujarat; Miller 1981; Rosenzweing and Schultz 1982; Dyson and Moore 1983; Kishor 1993).

**Table 2:** **Sex ratio at birth 1982–2000; 2000-2009**

**INDIA**	
1982-1984	**911**
1984-1986	913
1986-1988	912
1988-1990	911
1990-1992	900
1992-1994	885
1994-1996	884
1996-1998	902
1998-2000	900
2000-2002	892
2005-2007	914
2006-2008	904
2007-2009	906
2008-2010	905
2009-2011	906
2010-2012	908
2000	**886**
2001	875
2002	872
2003	868
2004	872
2005	876
2006	891
2007	903
2008	904
2009	898
2010	857

***Source:*** *Sample registration system- statistical report- 1982–2002; SRS statistical report 2012.*

*Annual report on vital statistics of india based on civil registration system- 2010.*

**Table 3:** **Overall sex ratio, child sex ratio 0–6 years, sex ratio 7 and + years - 1991-2001-2011**

	**1991**	**2001**	**2011**	**1991**	**2001**	**2011**	**1991**	**2001**	**2011**
**INDIA**	927	933	940	945	927	914	923	934	944
Andamane	818	846	878	973	957	966	790	831	868
Andhra P.	972	978	992	975	961	943	972	981	997
Arunachal P.	859	901	920	982	964	960	829	878	913
Assam	923	932	954	975	965	957	910	929	953
Bihar	907	921	916	953	942	933	895	914	912
Chandigarh	720	773	818	899	845	867	772	767	812
Chhatisgarh	985	990	991	984	975	964	986	992	995
Dadra Nagar	952	811	775	1013	979	924	937	779	752
Daman&Diu	969	709	618	958	926	909	971	682	589
Delhi	827	821	866	915	868	866	810	813	866
Goa	967	960	968	964	938	920	967	964	973
Gujarat	934	921	918	928	883	886	936	927	923
Haryana	865	861	877	879	819	830	826	869	885
Himachal P.	976	970	974	951	896	906	980	980	983
Kashmir	896	900	883	ND	941	859	ND	884	887
Jharkhand	922	941	947	979	965	943	908	935	948
Karnataka	960	964	968	960	946	943	960	968	971
Kerala	1036	1058	1084	958	960	959	1049	1072	1099
Lakshadweep	943	947	946	941	959	908	943	946	951
Madhya P.	912	920	930	941	932	912	905	916	933
Maharastra	934	922	925	946	913	883	931	924	931
Manipur	958	978	987	975	957	934	955	977	995
Meghalaya	955	975	986	986	973	970	947	971	989
Mizoram	921	938	975	969	964	971	911	930	976
Nagaland	886	909	931	993	964	944	865	890	929
Orissa	971	972	978	967	953	934	972	976	985
Pondicherry	979	1001	1038	963	967	965	982	1006	1047
Punjab	882	874	893	875	798	846	883	888	899
Rajastan	910	922	926	916	909	883	908	923	935
Sikkim	878	875	889	965	963	944	860	861	883
Tamil Nadu	974	986	995	948	942	946	978	993	1000
Tripura	945	950	961	967	966	953	940	945	962
UttarPradesh	876	898	908	927	916	899	863	894	910
Uttaranchal	936	964	963	948	908	886	933	973	975
West Bengala	917	934	947	967	960	950	907	929	946

***Source***: *Census of India 1991-2001-2011.*

**Table 4:** **Sex ratio at birth, 2000-2010**

	**2000**	**2001**	**2002**	**2003**	**2004**	**2005**	**2006**	**2007**	**2008**	**2009**	**2010**
**INDIA**	**886**	**875**	**872**	**868**	**872**	**876**	**891**	**903**	**904**	**898**	**857**
Andamane & N.	946	968	927	1004	935	967	933	949	961	973	934
Andhra Pradesh	936	934	936	973	972	987	973	974	986	985	983
Arunachal P.	728	887	806	812	864	799	792	854	875	806	806
Assam	n.a.**.**	n.a.**.**.	n.a.**.**.	750	n.a.**.**.	732	876	834	853	931	n.a
Bengala	932	904	882	889	903	906	890	918	914	929	931
Bihar	n.a.**..**	984	779	826	801	793	n.a.**.**	n.a.**.**.	n.a.**.**	715	n.a
Chandigarh	751	771	827	807	786	826	860	860	872	867	878
Chhatisgarh	933	925	920	926	919	939	919	919	886	924	906
Dadra Nagar	903	865	833	932	879	918	895	917	946	928	905
Daman & Diu	897	860	876	915	906	869	847	908	916	934	912
Delhi	820	809	831	823	823	822	831	848	1004	915	901
Goa	918	919	929	931	931	931	963	947	931	921	908
Gujarat	816	802	826	835	824	846	972	879	883	905	902
Haryana	792	789	829	814	796	827	857	860	854	853	838
Himachal P.	728	887	806	812	864	799	792	854	875	806	911
Jammu&Kasmi	882	876	865	865	869	873	878	878	892	896	917
Jharkhand	n.a.**.**	n.a.**.**.	n.a.**.**.	n.a..	840	830	835	865	878	864	865
Karnataka	955	971	956	924	858	943	952	1004	1047	1011	1025
Kerala	998	968	944	951	946	949	955	944	952	942	939
Lakshadweep	907	908	968	991	843	869	899	911	934	866	1015
Madhya P.	869	841	823	841	864	855	855	905	913	938	922
Maharastra	847	819	826	808	811	n.a.**.**.	n.a.**..**	n.a.**.**.	870	868	854
Manipur	914	753	949	923	1004	1011	963	913	950	934	770
Meghalaya	934	942	976	875	1011	918	965	864	959	948	912
Mizoram	911	927	913	926	945	940	952	938	942	949	948
Nagaland	820	894	980	971	977	980	804	823	802	883	871
Orissa	947	939	988	950	927	934	927	919	935	925	911
Pondicherry	942	946	937	942	936	950	955	950	923	927	917
Punjab	747	754	777	789	794	790	813	820	820	822	824
Rajastan	821	825	806	833	820	805	828	832	785	861	839
Sikkim	973	936	1252	935	956	954	941	981	951	955	961
Tamil Nadu	922	929	926	920	936	935	939	935	928	933	935
Tripura	931	914	1121	1202	n.a **r.**	n.a.	n.a	n.a a.	n.a..	n.a.	996
Uttar Pradesh	n.a**.**	n.a.	n.a.	n.a.	n.a	n.a	n.a.	n.a	n.a	n.a..	n.a
Uttaranchal	n.a**.**	n.a	n.a	n.a	845	864	873	869	860	855	858

***Source:*** *Annual report on vital statistics of india based on crs- 2009 and 2010.*

Although there are no official statistics on female suppression through infanticides, studies by local non-governmental organizations and national reports indicate that hundreds of thousands of baby girls disappear annually and are subtracted from the actual quota of female births. These female infanticides (although prohibited by the *Female Infanticide Prevention Act* in 1870) occur before births are duely recorded. Because they are missing at birth, these girls are, therefore, never counted so the *sex ratio at birth* deviates from the norm (which is normally 95 females born per 100 males born) in favor of male children. Far from being a “natural” trend, it is therefore caused by an “unnatural” gender selection system (Hvistendahl 2011). This system includes not only female infanticides but also more recent female feticide practices through sex-selective abortions facilitated by an increased availability of new technologies (prenatal ultrasound scans and foetal DNA testing) which, detecting the sex of the baby, enable early elimination of a female foetus. Health and social workers indicate that sex-selective abortion is such a widespread practice in all of the Indian states that is has become a *new* form of discrimination – a *modern surrogate* of female infanticide (Fraschetti 2011, pp.15-17)^h^. This is one of those cases where tradition and modernity are in a relationship of *mutual reinforcement* rather than mutual exclusion (Gusfield 1967). Modernity is accepted from tradition and set at the service of traditional cultural orientations, which therefore, in turn, ensure its development. These procedures are, in fact, especially widespread among the low and middle, more urbanized, castes due to the *sanskritization process* and *hypergamy* custom. The practice of marrying lower status women to higher status men within the same caste, limits the number of potential husbands available above the lower family status. This situation of *marriage squeeze* for women (Bhat and Halli 1999) intensifies the competition for suitable grooms and requires therefore the cost of a considerable dowry to the bride’s family so as to ensure her a suitable husband. The Dowry system and subjugation of the bride’s family to the groom’s family as effect of matrimony explains recourse to female infanticide and feticide (the more daughters the more dowries, and the more weddings the more subservience) and, along with them, the gender imbalance at birth. Even though a law was introduced in India in 1996 (the *PNDT Act* - *Pre-natal Diagnostic Techniques Act*) which prevented the knowledge of unborn child gender through ultrasound scanning and banned abortions of healthy female fetuses, the recourse to selective sex abortions is currently such that the use of the gendercide term can be justified. Also due to inadequate and ineffective government control in the actual application of the law, during the last thirty years – from 1980 to 2010 - a total of 4.2 to 12.1 million female fetuses were first “selected “and then aborted illegally (primarily involving second and third offspring; Jha et al*.*^2011^). It is still in the Northern States that female feticide as well as infanticide is particularly widespread – in Punjab, Haryana, and in Uttar Pradesh, the so called “*Bermuda Triangle*”, girls frequently go missing (Agnihotri 2000). The feticide capital of India is, in fact, the Jhajjar district, in the Northern State of Haryana. Here there is a sex ratio at birth of 774 girls per 1000 boys. Whereas lower status families tend to make more use of infanticide, higher status families prefer sex selective abortions. Dowry duties remaining constant, the increase in average income followed to economic modernization has evolved into greater access to prenatal diagnosis and has actually maintained the gender gap instead of reducing it. Considering that a daughter is anyway a financial burden, the more Pre-natal determination is, in fact, considered a sort of “investment” for the family of origin (*Action Aid Report* 2009).

Indian female deficit reflects therefore the strong traditional Indian preference for sons. First of all, only sons can inherit the family name and property. Secondly, sons are the main financial support once the parents grow old, as these countries follow the patriarchal residence tradition. Therefore, after marriage daughters leave their parents’ home while sons bring their wives into the family residence. In the third place, the daughter’s marriage is much more expensive than the son’s because in the former case a dowry must be paid for and given, while in the latter case the son takes the dowry from the wife and her family. And lastly, *only* sons are allowed to celebrate ancestral rites after the father’s death.

**Figure 3:**
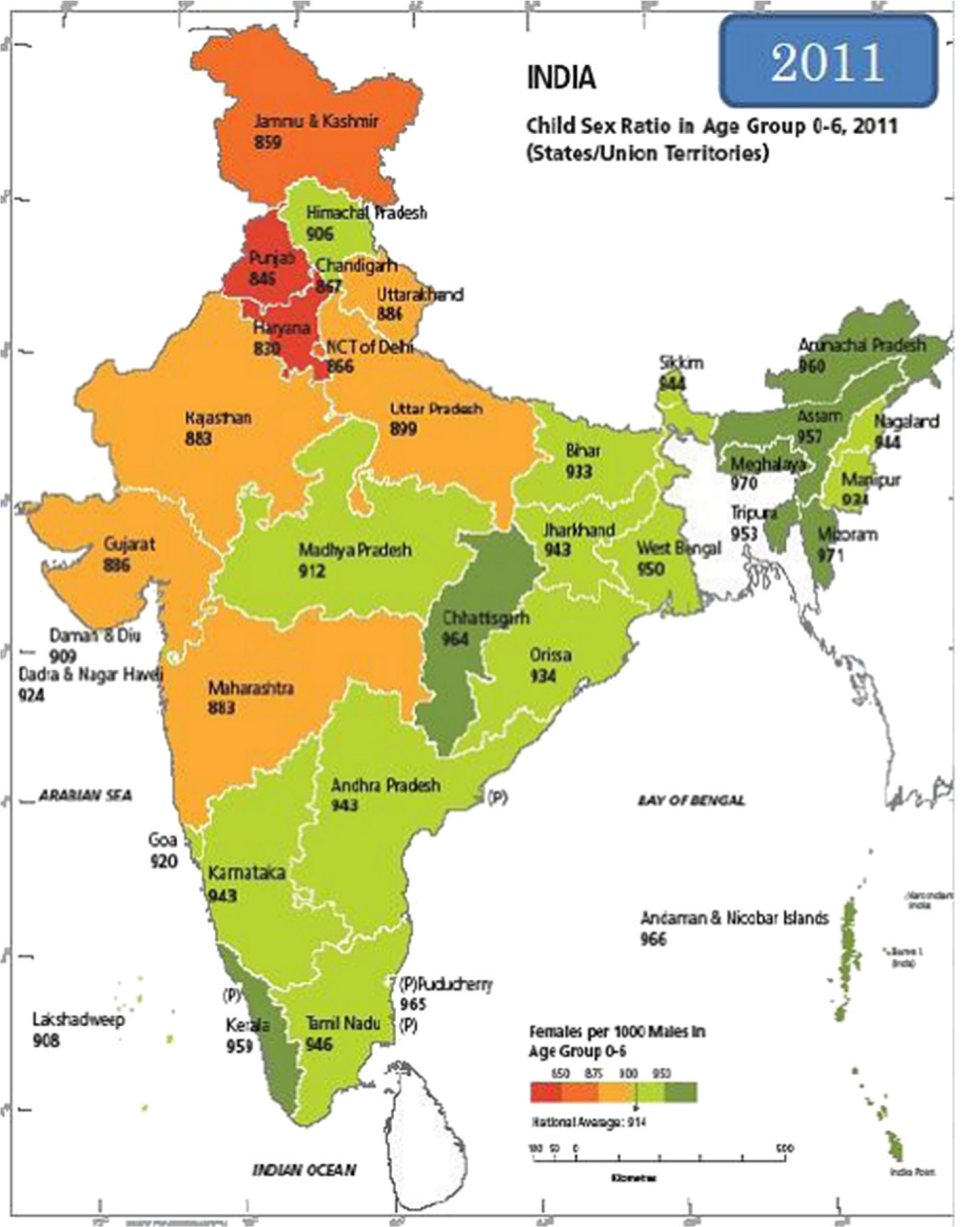
**Child sex ratio 0–6 years, 2011.** Source: Census of India 2011.

This preference produces forms of gender discrimination that affect women’s health and survival in many ways, influencing not only reproductive behaviour but levels of nutrition, education (with an overall gender gap in a 16.68% literacy rate and a higher local gender gap in the North; *Census of India* 2011) and health care for girls particularly in the first years of life (*The World’s Women* 2000). This gender gap in childhood investment especially relating to girl child healthcare (gender gap in medical care, vaccinations, medical expenses, and illness treatment) has been considered the most direct determinant of female child post-natal mortality (significantly greater, again as always, in the Northern Regions Arokiasamy 2004; Bhattacharya 2006; Oster 2009). Oster, for instance, using nation wide data, concludes that vaccination explains 20%-30% of excess female IMR, malnutrition 20%, and lack of illness treatment (diarrhea and respiratory ailments) explains another 4%. The result is that India is one of the countries with the highest gender difference in infant mortality rate: Indian girls aged 1–5 have a 75% higher mortality rate than their male counterparts (United Nations-Department for Social and Economic Affaire 2011). From 1985 to 2005, circa 1,200,000 girl children died during their first months and another 1,800,000 died before their 6^th^ birthday (*Archives of Pediatric and Adolescent Medicine* 2011). Pre-natal and post-natal discrimination, therefore, complementarily contribute to gender imbalance in the sex ratio. Considering female post-natal infant mortality as another significant indicator of gender discrimination in India, the 0–6 and even the 7 and over years *sex ratio* is consequentially unfavorable regarding women and again, above all, in North India (Table 4; Figure 3). According to data from the 2011 Indian Census, here, preference for male children has caused a decline of 914 girls per 1,000 boys, the worst result in the last century. In 2001, females under 5 were actually 927 out of 1,000 males.

In conclusion, it has been estimated (Guilmoto 2005, 2012) that in Asia, between China and India, if the rapport between males and females were to have remained at a natural level, the continent would have had about 160 million more women, with India missing approximately 10 million. According to estimates by the United Nations, in the last 20 years, up to 200 million women and girls are demographically missing in the world. This figure is really too high to be attributed to biological causes alone. These *missing women*, as Sen (1992) calls them do not signal the emergence of a strange biological mystery. There is no biological mystery to be solved here but rather the adamant persistence and viscosity of social and cultural factors which use modern progress at own advantage.

### From *missing women* to *dowry uxorcide*: a *mutual reinforcement* between modern materialistic culture and traditional dowry practice

Dowry practice is an *intrinsic* tradition of Indian society, spread throughout the entire territory, with stronger consolidation in the North. The more women are lacking in producing wealth, the more dowry practice becomes instrument to make desiderable an unproductive asset so as to get rid of daughters as soon as possible through marriage. In addition, as already seen in the previous section, the institutionalization of the *sanskritization process* and *hypergamy custom* justified in the frame of Hinduism reinforce the reasons for the dowry practice persistence. Daughters are, therefore, burdens on their family of origin in many ways. The dowry obligation is consequently the direct cause of infanticides and feticides, physical and psychological domestic violence, arranged child marriages, induced suicide and uxoricides, all fully recognized and grouped under dowry related violence (De Somviele and Marchand 2003). According to official statistics from the *National Crime Records Bureau,* just in April-November 2009 alone, there were about 11,004 cases of extreme-violence against women. Reports of rape, molestation, dowry death rose by 6.4 percent in 2012 from the previous year, with the highest number of rapes recorded in the capital city. Although more women, nowadays, denounce violence, the figures indicate a general rise of gender related violence in India. Nevertheless, these results represent only the tip of the iceberg which remains largely hidden and out of official surveys.

Banned in 1961 for the above reasons by the *Dowry Prohibition Act*, the dowry still remains an *institution* in contemporary India. In spite of modernization and women’s increasing role in the market economy, dowries are still expected by grooms and their family. Moreover, the demanded economic amount is also increasing. Interestingly, in fact, modernization has only not erased the dowry tradition as one would normally expect but has reinforced it in so far as it produces an increasingly materialistic culture that, increasing the desirability of consumer goods, in turn, has increased the value of the money and goods that dowries provide. In fact, in time, by a mounting consumerism, the demand for material goods that may be acquired via dowries has increased and has become an easy way of enrichment, a way to obtain money and material goods of any kind: from the latest modern TV set to the newest model of mobile phone or car and motorcycle. According to Srinivasan and Lee (2004), the Indian dowry system should not be viewed simply as a traditional practice that will eventually be eliminated by modern processes of social change, but “rather as an important component of a marriage system that is changing in response to a progressively more materialistic culture” (2004, p. 1115). From this perspective, the dowry practice is flourishing because young married couples, even the so called more modern ones, may see the dowry as a good way of obtaining consumer goods. So the dowry practice persists and continues to be an influential part of the Indian mate selection system with several adverse consequences for women. When dowry expectations are not fulfilled, wives may suffer severe consequences, including physical abuse and death.

There are many reports of violence against women whose dowries are deemed insufficient by their husbands or the husbands’ families (i.e. Mandelbaum 1999). Dowry expectations do not end once the wedding has been celebrated. It is tacitly understood that the bride’s family must continue to provide goods for the entire duration of the conjugal relationship. Lack of compliance to ever greater demands, exposes the bride to retaliation from the groom, which explains the unusual frequency of domestic violence and dowry deaths regardless of economic and social strata. Even though the Indian Penal Code, after feminist movement pressure, in 1986 strictly regulated the matter of dowries for deterrent purposes, there were about 8,391 cases of dowry related deaths in 2010 alone, according to data from the *National Crime Records Bureau.* In 2012, there were 8,233, nearly one woman per hour. In spite of modernization and probably favored by it, the incidence of dowry deaths grew by nearly 3% over the previous five years. Very often, these crimes go unpunished and classified, because of false witness of husband’s family, as domestic accidents or even suicides cases.

### Female widowhood in India. The *new Sati:* a look from the perspective of a Goffman de-personalization process

The *Manu Code* regulates women’s position within marriage and especially within widowhood. In this latter case, Hinduism provides one more reason, a very strong reason to support the patriarchal orientation and consolidates it by appealing to divine authority. There is no other virtue that the community attributes to women, no other existential reason, no other identity than absolute devotion to husband in life and after his death. The ideological justification and the normative cogency or institutionalization of husband devotion (or *patryvata*) goes back to the sacred and to the principle of the relationship between *dharma* and *reincarnation*. Religious sanction in case of disobedience to the duties of chaste and faithful wife before and after the husband’s death (endless cycles of reincarnation into vile beasts)^i^ and the collective contempt from the community ensure women’s social compliance.

According to 2001 *Census* Data, India had 34 million widows, 8 million more than in the 1991 *Census*; whereas, according to recent findings, there are around 40 million, about 8% of the entire Indian female population (*Census of India 2011*). If it is true that the number of widows rises as age increases, the 2001 *Census* (latest available figures) shows a consistent number of young widows aged 15–24 from 1971 to 2001. The 1991 Census showed that the 15–19 year old bracket had a total of 65,590 widows with as many as 28,828 10–14 year olds (*Census of India* 1991). In 2001 there was 0.68%, of young widows out of the 20–24 year old female population and 0,20% out of the 15–19 year old female population (Table 5). The persistence of arranged child marriages practice (outlawed in 1929 by the *Child Marriage Restraint Ac*t) due to the same reasons inducing the family of origin to get rid of an unsustainable burden as soon as possible, the consequential great age difference between the spouses, polygamy and the persistence of taboo against widow’s re-marriage (although allowed today by law) all contribute in explaining the quota of very young widows found^j^.

**Table 5:** **Indian widow distribution by age bracket in 1971, 1981, 1991, 2001 – percentage out total female population of the respective age bracket**

**Fasce d’età**	**Vedove**			
	**1971**	**1981**	**1991**	**2001**
**15-19**	0,34%	0,21%	0,18%	0,20%
**20-24**	0,9%	0,69%	0,59%	0,68%
**25-29**	1,85%	1,5%	1,15%	1,37%
**30-34**	3,95%	3,12%	2,32%	2,59%
**35-39**	6,99%	5,42%	3,94%	4,45%
**40-44**	14,19%	10,86%	7,71%	7,71%
**45-49**	20,42%	15,96%	11,16%	11,31%
**50-54**	36,55%	29,72%	21,93%	20,18%
**55-59**	41,07%	31,79%	23,76%	21,95%
**60-64**	62,55%	56,07%	45,24%	40%
**65+**	74,11%	70,34%	59,83%	56,62%

***Source:*** *Census of India, marriage statistic* 2001.

Regardless of age, husband devotion also after his death means for widows, as prescribed more than 2000 years ago in the sacred text of *Manu,* a life made up of social and economic deprivation. Widows’ social death replaces today the *sati practice* (sacrificial immolation of widows on the husband’s funeral pyre) which was banned by British colonial law in 1829–1830 and effectively eradicated in the late 1880s. Beyond any religious justification, the various strategies of social contempt and marginalization used from community against widows (in addition considered guilty to have deserved their widowhood status because of some “sin” they have committed in the previous life) are aimed at solving the problem of their social sustainability. Once the husband dies, the widow becomes a heavy burden on the husband’s family, a threat for the inheritance and, therefore, an *unwanted insider* (Srivastava 2002). Hinduism, for its part, ends up by religiously legitimizing every form of widows’ social exclusion preserving gender social order. This explains why traditional *sati (altruistic suicide* in Durkheim’s classification), although illegal, has still not entirely disappeared from India. In the nineteenth century, especially in Bengala, this phenomenon reached exorbitant figures: from 1815–1827 about 5,388 cases of *sati* were recorded (Piretti Santangelo 1991). There were obvious economic interests behind these alarming numbers. In fact, Bengala applied the Dāyabhāga system whereby widows could inherit their husband’s property. The *Bengal Sati Regulation Act* in 1829 made widow immolation illegal and more adequate government controls have significantly decreased the number of sacrifices. Yet, still today in North India (Bengala, Rajasthan, Madhya Pradesh, Uttar Pradesh, Chattisgarh), in the most rural and inner areas of the country, evidence of this ritual is found.

Nowadays, as was said above, the widowhood implies the social death rather than the physical death. The *new sati* requires the widows to withdraw from the world, a renouncing of one’s own life by living in a state of deprivation and humiliation or in a state of *social invisibility* and *de-personalization*^*k*^*.* Therefore, the strategy changes but not its symbolic and functional social significance. For the widows who remain within the husband’s family, which happens in the majority of cases in the South, but nevertheless in a condition of severe social and family marginalization which even prohibits physical contact because widows are considered untouchable and inauspicious, there are other widows who live by begging and praying in the temples and *ashrams* of Vrindavan or Varanasi, the two Uttar Pradesh cities where widows are most likely to be abandoned by their husband’s family. It is significant to note that Vrindavan or Varanasi widows come above all from the North, West Bengala, Rajasthan, Bihar, Uttar and Andhra Pradesh (Chen 1998; National Commission for Women 2009- (NCW-Report))^l^. Specific behavior, feeding and dress codes (little or poor and standardized feeding, strictly subject to fasting regulations and, above all, devoid of any appealing element, which surrogates physical death) have the function to annul widow identity in order to stifle any potential social or sexual claim. These *self reduction* practices enter into mechanisms whose effectiveness has been pointed from sociological theory. To the extent that people invest a sense of self in what they posses, these mechanisms consist in the systematic loss of any personal possession emphasizing individuality, including own name (Goffman 1961). The systematic process of de-personalization which widowhood implies for women consists as well in total loss of the woman’s usual look (the personal façade) which is sacrificed to homologation criteria such as shaving of the head, erasing the *bindi*, breaking the *bangles*, the white *sari* imposition, the losing the female pronoun *she* which is substituted by the neuter pronoun *it,* highlighting a no longer acknowledged social identity. In the end, widows are mutilated in their identity, de-sexualized, no longer individuals but things, non-entities – nothing, “confined within a circle of the zero” (Chakravarty 2002, p. 255), marginalized, “tolerated but never welcomed” (Khanna 2002, p. 29).

Thus, while the government has failed in checking the phenomena, society has succeeded in placing the widow at its edge, putting her in a condition of being physically alive but socially dead and ultimately institutionalizing her social marginality.

## Conclusions

This work has amply shone how the social existence of women in India is exposed to serious risks from the moment of conception by virtue of a combination of patriarchal and an economic-cultural legacy from the past and present day modern technological and cultural elements, closely interweaving and mutually nourishing each other. There is little evidence that women’s power is increasing in India. Instead, Indian culture remains broadly patriarchal, undermining women’s status, also due to unexpected mutual adaptations and reinforcements of modernity and tradition.

These unexpected combinations are not comprehensible in light of a linear concept of social change which is founded, in turn, on a concept of social systems as interaction systems that relate to exterior or environmental perturbations according to proportional cause and effect relationships. According to this approach, based on the classic scientific epistemological Newtonian.-Laplacian paradigm, viewing social systems as systems characterized by a linear structure of interaction relationships among system’s components/agents means assuming that evolution or change processes are linear as well, and therefore, their predictability and control are ensured (see Condorelli 2013). From this perspective, therefore, facing modernization inputs, behavioral attitudes and interaction relationships should be less and less regulated by traditional values and practices as exposure to modernizing influences increases. And progressive decreases should be found in rates of social indicators of gender inequality like dowry deaths (the inverse should be found in *sex ratio* trends). However, this linear, proportional weakening process of tradition as a regulating principle of social interaction was not confirmed from data. As we have seen, modernity and tradition reinforce each other reciprocally. Tradition absorbs, combines with modernization and, rather than weakens until it disappears, strengthens, continuing to last in regulating social interaction relationships and harnessing the potential for social change of the modernization process. This is the case of female infanticides and feticides and dowry practice, which has extremely serious adverse consequences for women.

One would normally expect traditional attitudes toward the dowry system, along with arranged marriage customs to be *a function* of exposure to modernizing influences that would reduce support for it. According to a linear approach, one would, that is, expect a proportional weakening of these traditional practices as modernization increases. However, although some dimensions of modernization such as education, urban residence, and exposure to modern media may diminish support for the dowry (favouring an exposure to modern ideas that challenge conventional thinking, customs and lifestyles), arranged Indian marriages and the accompanying dowry system have proved to be extremely resistant to social change. On one hand, as we noted, regardless of the persistence of structural economic conditions making women an economic burden for family of origin, the *Sanskritization process a*nd the *Hypergamy custom,* with a decreased fertility among higher status families, increase economic competition for desirable husbands for daughters and continue to make the dowry practice necessary for successful mate selection. On the other hand, some reasons for this practice’s lasting hold are connected to modernization itself. Dowry practice seems to have found in modernization further reasons for its reinforcement. The modernization is helping to produce and increment materialistic culture, which in turn increases the desirability and the value of consumer goods that dowries provide. So, still today the dowry is viewed as a easy way of obtaining wealth and continues, therefore, to be an influential part of the Indian mate selection system in response to a progressively more materialistic culture, in spite of its problematic consequences for women. This leads, therefore, to the conclusion that, as Srinivasan and Lee observed (2004), the Indian dowry system should not be regarded simply as a traditional practice that will eventually be eliminated by processes of social change as modernization progress. On the contrary, as mounting consumerism of Indian culture increases for material goods that may be acquired via dowries, it is not yet clear that modernization will negatively affect the dowry system. This practice may be quite resistant to change because it carries tangible benefits in an increasingly materialistic culture.

These unexpected processes do no other than confirm the need for a new theoretical and methodological approach toward social systems study. This new approach seems to be more and more validly identified within the conception of social systems as complex systems. The new complex system approach has been formulated in the last decades starting with natural sciences and established in social sciences as well. Treating all systems (natural and social systems) as complex systems seems to be an efficacious substitute for the classic linear approach. From this perspective, social systems, like natural systems, adapt to disturbances from the outside and self-organize as a result of non-linear interactions among components of the system, expressing non proportionality between cause and effect. For this reason, on the whole, social system can produce new and unexpected orders, *new*, *unexpected, surprising* and *unpredictable* patterns of regulation of social interaction or *emergent effects* (see Condorelli 2013). Within the framework of an *emergentist conception* of social change, the progressive weakening of tradition up to its disappearance becomes therefore only one of many possible scenarios, but certainly not the only one. That is, only within the framework of *emergentist theory* of social change is it possible to understand the lasting strength of the patriarchal tradition and its problematic consequences in a society with features of undoubted modernity.

## Endnotes

^a^Even though it is not formally acknowledged, castes still continue to have a strong cultural and social valence.

^b^The *sanskritization process* is the process whereby low or middle Hindu caste classes try to raise their social position by adopting the rites, customs and beliefs of the higher positioned castes and through marriage of daughters to higher status men within the same caste.

^c^The central part of the *Manu Code* (the *Manava Dharma Shastra)* extensively regulates individual and social life, treating duties and privileges of the castes, duties of women as mothers, wives and widows, ritual bylaws, marriage conditions, economy, government, children’s education, what constitutes pure acts, regulating relationships between castes, and impure acts (for example, affronts or harm caused by inferior castes against higher ones or by women or widows against their husbands) which are punished by infinite rebirths as abject creatures.

^d^Sociology theory has outlined a complex interweaving of relationships between structures and superstructures. The first relationship is that of *necessary determination from structure to superstructure,* as in the Marxian approach, whereby a particular economic structure determines power relationships and at the same time a superstructure that serves to maintain and strengthen them. Secondly, there are *mutual autonomy* relationships, as Durkheim points out, where, although the equivalence *type of society* = *type of religion* is accepted, the superstructure is not merely a simple epiphenomenon of the structure, constituting a *sui generis* reality with its own autonomy and independence. The third type of relationship is *mutual conditioning* where the superstructure seems able to, in turn, determine structure as Weber demonstrated by studying the relationship between Protestantism and Capitalism and disputing Marxist historical materialism in favor of a relationship of mutual influence. All depends, in fact, upon the point of view from which we study these relationships. Even Parsons, as we know, concords with Weber’s approach.

^e^In the Vedic period, dating from 2500 to 500 B.C., women possessed the same rights and privileges as men as far as education, work, matrimony, children’s upbringing, religious participation, and access to Veda study facilities. They were able to reach high levels of spiritual elevation and become sources of wisdom for the community (*rishi*). Their role in the family and in society, therefore, was considered fundamental. Dowries were not common, but requested only if the bride had a physical defect. *Sati* was not widespread and widows were respected, being able to remarry if they so chose. From 500 B.C., when the Vedic golden era of Hindu civilization declined due to famine and plagues, and above all due to the Persian and Doric invasions, Vedic principles of equality among the sexes disappeared as well. Because of economic change and territory defense exigencies, there was a social de-valorization of women and a growing, consolidating preference for male offspring dominance. Casteism, which views women as relegated to the lower caste of *Sudhra*, was established during this time, while the caste of priests and warriors were placed on top of the caste hierarchal scale. A fundamental aspect of the decadence of women’s condition was the loss of religious prestige. Along with the institution of the Priestly caste (*Brahman*) women were denied the right to read the Veda and to attend and celebrate religious rites (such as parents’ funeral rites, prerequisite for liberating from *transmigration*, accorded only to male offspring), and the entire religious and political (caste) set up of society was established on male superiority. Even high caste women were not allowed to directly participate in religious rites. Women’s freedom, also partly due to invasions, was prone to strong restrictions. Their social role was confined to their hearth; their education was purposely limited to further reinforce female subordination. Weddings became rigorously endogamous, decided by the family at a very early age; extra marital affairs were punished by expulsion and often by death; dowries became obligatory, and the custom of *sati*, prohibiting widows from remarrying, was consolidated. This theoretical nucleus of Hinduism is still very pervasive to the present day, even if it is modulated differently in the different regions of the country according to the varying production activities and according to the amount of female participation in these activities.

^f^The idea according to which *Truth*, everything that is worthy of a human being, is *God* is the theoretical nucleus of Gandhi reform. It is a revision process and spiritual and moral renewal of Hindu tradition, concerning every mortifying aspect against human dignity. Revision of caste theory and the affirmation of gender equality inspire, in fact, the Ghandian Illuminism. Continuous quest for the Truth, which means giving worth to individual freedom compared to the community and to whatever concept of the *common good,* is according to the Mahatma the only true path for a society based on tolerance, non-violence, mutual respect, equality and human freedom.

^g^There are slightly fewer women than men in the world- 99 women per 100 men. On average, men outnumber women in most of Asia and in North Africa. Confirming, in fact, the trend found in 2001, in 2011 in Europe and in the United states there was an average of 106 females per 100 males; in Latin America there were 101; in Sub-Saharan Africa, 103, in North Africa, 99 and in many countries in Asia, 95, with 93 for China and 94 for India (*Statistics Division of the United Nations Secretariat from women’s indicators and statistics database* based on *Population Division of the United Nations Secretariat, World Population Prospects* 2011). The reason for this trend is that in many regions of undeveloped nations, for certain age brackets, in particular, the 2–30 year olds, female mortality exceeds male mortality. It is not due to biological factors nor to economic underdevelopment but to social and cultural factors, namely to the socio-cultural value of women depending on whether they participate or not in production of wealth. African Sub-Saharan Countries and generally those in West Africa, although greatly underdeveloped, have in fact an excess of women. In these countries, women have an active role in the production of wealth and goods in so far as agriculture is carried out for the most part by women, with the help, in part, by men (*Female Cultivation System*). Not being an economic burden but an asset, brides’ family need not prepare dowries for marriage but it is the groom that has to pay the *bride price.*

^h^Infanticides as well as abortions are not allowed in the Hindu religion; some sacred texts like the *Satra*, in fact, openly condemn these types of practices. Even though female infanticide and sex selective abortion were prohibited (the *Female Infanticide Act* or *Act VIII* in 1870, instituting repressive, penal and civil, measures in order to prevent female infanticide, and the *Pre-natal Diagnostic Techniques Act* in 1996) they have continued to be practiced due to the tradition of dowries and the *sanskritization process* (see note 3) involving marriage of daughters to higher status men within the same caste. On one hand, this bring wealth to the family receiving the dowry, but, on the other hand, it leads to a financial drain for the family who must prepare the dowry and encourages possible infanticides. Social and family pressure on the “guilty” mother because she was unable to give birth to a son, is so great that, despite the religious prohibition, female infanticide and sex selective abortion is the only means for “reparation” and the way to avoid social and family marginalization which can actually endanger the woman‘s life. As several national and international reports denounce, the choice of female infanticides and feticides is a “forced” one, and a compassionate act in view of the fate awaiting the unborn female child just because it is a woman (*Action Aid Report* 2009).

^i^According to the *Manu Code,* “A widow should be long suffering until death, self-restrained and chaste. A virtuous wife who remains chaste when her husband has died goes to heaven. A woman who is unfaithful to her husband is reborn in the womb of a jackal” (*The Laws of Manu*, Chapter 5, verse 156–161).

^j^Although the *Child Marriage Restraint Act* (modified in 1978) prohibits marriage with children, establishing the minimum age at 18 for women and 21 for men, this law remains very far from being applied. Today, child marriages are still being celebrated in some parts if India, such as in Rajastan in the North. On *the Akha Teej* and *Basant Panchmi* festivals, thousands of children get married. Little 8–10 year old girls are obliged to marry men, older than them by 20, 30 or even 60 years who for obvious reasons will soon leave them widows. This is an unspoken tragedy, according to UNICEF which estimates that about 47% of all child marriages worldwide in 2011 took place in India.

^k^The widow’s social death is characterized by certain visible features which, depriving her of her femininity, serve to identify her new status compared to her former “wife” status : the white *sahari*, the color of mourning and asexuality, rather than the red one, symbol of fertility, the shaving of the hair (act of symbolic femininity castration), the removal of the *bindi,* the typical red spot on the forehead which means the woman is married and likewise, the *sindur*, the carmine red paste smeared between the forehead and the hairline, the breaking of her bangles or colored glass bracelets, and the absence of any form of ornament, embellishment or make up. There are, in addition, various food restrictions (such as eating only once a day, the interdiction from eating sweets and the obligation of frequent fasting), the loss of personal goods, the interdiction from participating in any religious rite and ceremony and accessing any medical care whatsoever. Tradition dictates that a widow cannot remarry, nor return to her paternal family, nor to a normal social life. Even living in the home of her deceased husband’s family means being relegated to a condition of near slavery and servitude. The widow becomes an economic “burden” and is considered inauspicious; she will have to serve her in-laws her entire life, having to support insults, harassment or mistreatment by the men of the household and by the mother-in-law due to dowry related problems. Women in the course of their life time, therefore, pass from a despot father/husband to a tyrant-mother-in-laws (Fraschetti 2011). In this sense. for widows *life* in India is even worse than *Sati* (Piretti Santangelo 1991).

^l^The last *NCW* Report, commissioned by the Indian Supreme Court (*National Commission for Women*) dates back to 2009. Its aim was to explore the situation of women and the thousands of widows who throng the holy city of Vrindavan. Of the 216 women interviewed, widows (as much as 78% of the sample) lived in temples and in ashrams, forsaken in Vrindavan and left in miserable conditions by the deceased husband’s relatives or their own children. About 30% had resided their for over 20 years; 20% for 10 to 20 years and another 30%, for less than 5 years. In 74% of the cases, widows came from West Bengala, and their widowhood for the most part began in adolescence, age 15–16, after a child wedding (even as young as 10 or 12) with men at least twice their age. Two out of five widows got married before the age of 12 and almost one out three was widowed by the age of 24. Moreover, it is estimated that in the Subcontinent, one out four women marry before the legal age of 18, and almost one out of five marry under the age of 10. Widows generally live in very oppressive conditions, depending mainly on begging and prostitution for survival (*NCW Report* 2009).

## Additional file

Additional file 1:
**A.** **Table A Percent distribution of the de jure population according to wealth, quintile/rural and urban population, NFHS-3, India, 2005–2006.** **B.** Hinduism codified a conception of femininity of which the most systematic exposure can be found in the Chapter 5 and 9 of *Manu Code.* They deal with duties of women towards husbands and enshrines male superiority over women.
