# Supplementary material for: An emergentist vs a linear approach to social change processes: a gender look in contemporary India between modernity and Hindu tradition
Source: Springerplus. 2015 Apr 1;4:156. doi: 10.1186/s40064-015-0933-7 (PMC4382501; doi:10.1186/s40064-015-0933-7)
Supplement: Additional file 1: — A. Table A Percent distribution of the de jure population according to wealth, quintile/rural and urban population, NFHS-3, India, 2005–2006. B. Hinduism codified a conception of femininity of which the most systematic exposure can be found in the Chapter 5 and 9 of Manu Code. They deal with duties of women towards husbands and enshrines male superiority over women. [file 40064_2015_933_MOESM1_ESM.doc]

Additional file 1:**A. Table A.** Percent distribution of the de jure population according to wealth, quintile/rural and urban population, NFHS-3, India, 2005–2006.

|  |  | **Wealth quintile** | | |  |  |
| --- | --- | --- | --- | --- | --- | --- |
| **Residence/state** | **Poorest 20%** | **Second**  **20%** | **Middle**  **20%** | **Fourth**  **20%** | **Richest**  **20%** | **Total** |
| **India** | 20.0 | 20.0 | 20.0 | 20.0 | 20.0 | 100.0 |
| Urban | 3.0 | 6.4 | 13.8 | 28.9 | 47.9 | 100.0 |
| Rural | 27.7 | 26.1 | 22.8 | 16.0 | 7.4 | 100.0 |
| **North** |  |  |  |  |  |  |
| Delhi | 0.2 | 2.7 | 8.6 | 18.9 | 69.6 | 100.0 |
| Haryana | 4.1 | 12.6 | 24.6 | 27.8 | 31.0 | 100.0 |
| Himachal Pradesh | 1.2 | 8.8 | 24.1 | 30.8 | 35.1 | 100.0 |
| Jammu & Kashmir | 2.8 | 12.3 | 29.8 | 29.5 | 25.6 | 100.0 |
| Punjab | 1.4 | 6.3 | 15.3 | 28.8 | 48.1 | 100.0 |
| Rajasthan | 24.2 | 17.7 | 21.8 | 17.3 | 19.1 | 100.0 |
| Uttaranchal | 6.0 | 15.3 | 22.1 | 23.8 | 32.8 | 100.0 |
| **Central** |  |  |  |  |  |  |
| Chhattisgarh | 39.6 | 26.9 | 14.7 | 8.7 | 10.2 | 100.0 |
| Madhya Pradesh | 36.9 | 24.2 | 13.1 | 12.7 | 13.1 | 100.0 |
| Uttar Pradesh | 25.3 | 24.9 | 19.4 | 16.8 | 13.6 | 100.0 |
| **East** |  |  |  |  |  |  |
| Bihar | 28.2 | 29.2 | 18.7 | 14.6 | 9.4 | 100.0 |
| Jharkhand | 49.6 | 15.5 | 11.1 | 11.9 | 11.9 | 100.0 |
| O rissa | 39.5 | 19.9 | 17.3 | 13.4 | 9.9 | 100.0 |
| West Bengal | 25.2 | 24.4 | 18.7 | 17.8 | 13.9 | 100.0 |
| **Northeast** |  |  |  |  |  |  |
| Arunachal Pradesh | 21.1 | 25.6 | 20.8 | 16.1 | 16.4 | 100.0 |
| Assam | 19.8 | 30.7 | 22.6 | 15.0 | 11.8 | 100.0 |
| Manipur | 2.4 | 15.7 | 33.4 | 31.8 | 16.7 | 100.0 |
| Meghalaya | 11.3 | 21.8 | 26.5 | 24.0 | 16.4 | 100.0 |
| Mizoram | 2.5 | 6.1 | 19.2 | 33.4 | 38.8 | 100.0 |
| Nagaland | 7.8 | 22.6 | 28.9 | 25.7 | 15.0 | 100.0 |
| Sikkim | 1.9 | 10.6 | 22.9 | 31.7 | 32.8 | 100.0 |
| Tripura | 11.0 | 24.4 | 42.0 | 15.0 | 7.6 | 100.0 |
| **West** |  |  |  |  |  |  |
| Goa | 2.2 | 5.3 | 14.2 | 22.9 | 55.3 | 100.0 |
| Gujarat | 7.2 | 14.2 | 19.1 | 27.6 | 31.9 | 100.0 |
| Maharashtra | 10.9 | 14.9 | 17.4 | 24.3 | 32.5 | 100.0 |
| **South** |  |  |  |  |  |  |
| Andhra Pradesh | 10.8 | 17.6 | 29.2 | 25.4 | 17.1 | 100.0 |
| Karnataka | 10.8 | 22.2 | 24.0 | 23.2 | 19.8 | 100.0 |
| Kerala | 1.0 | 4.1 | 12.2 | 37.8 | 44.8 | 100.0 |
| Tamil Nadu | 10.6 | 15.6 | 29.9 | 24.4 | 19.5 | 100.0 |

The National Family Health Survey (NFHS) is a large-scale, nationally representative survey of Indian households including more than 85,000 households with an overall response rate above 98% and providing state and national-level estimates of key demographic, health and household socioeconomic indicators. NHFS will implement the fourth National Family Health survey in 2014–2015 (NHFS-4).

**B.** Hinduism codified a conception of femininity of which the most systematic exposure can be found in the Chapter 5 and 9 of *Manu Code.* They deal with duties of women towards husbands and enshrines male superiority over women. Below some exemplifying verses are listed:

Cap 5

154. Though destitute of virtue, or seeking pleasure elsewhere, or devoid of good qualities, yet a husband must be constantly worshipped as a god by a faithful wife.

155. No sacrifice, no vow, no fast must be performed by women apart from their husbands; if a wife obeys her husband, she will for that reason alone be exalted in heaven.

156. A faithful wife, who desires to dwell after death with her husband, must never do anything that might displease him who took her hand, whether he be alive or dead.

157. At her pleasure let her emaciate her body by living on pure flowers, roots, and fruit; but she must never even mention the name of another man after her husband has died.

158. Until death let her be patient of hardship self-controlled, and chaste, and strive to fulfil that most excellent duty which is prescribed for wives who have one husband only.

160. A virtuous wife who after the death of her husband constantly remains chaste, reaches heaven, though she have no son, just like those chaste men.

161. But a woman who from a desire to have offspring violates her duty towards her deceased husband, brings on herself disgrace in this world, and loses her place with her husband in heaven)

164. By violating her duty towards her husband, a wife is disgraced in this world, after death she enters the womb of a jackal, and is tormented by diseases the punishment of her sin.

165. She who, controlling her thoughts, words, and deeds, never slights her lord, resides after death with her husband in heaven, and is called a virtuous wife.

168. Having thus, at the funeral, given the sacred fires to his wife who dies before him, he may marry again, and again kindle he fires.

Cap 9

2. Day and night woman must be kept in dependence by the males of their families, and, if they attach themselves to sensual enjoyments, they must be kept under one's control.

3. Her father protects her in childhood, her husband protects her in youth, and her sons protect her in old age; a woman is never fit for independence.

14. Women do not care for beauty, nor is their attention fixed on age; thinking 'It is enough that he is a man,' they give themselves to the handsome and to the ugly.

15. Through their passion for men, through their mutable temper, through their natural heartlessness, they become disloyal towards their husbands, however carefully they may be guarded in this world.

16. Knowing their disposition, which the Lord of creatures laid in them at the creation, to be such, every man should most strenuously exert himself to guard them.

Doniger W, Smith BK (transl.) (1991) The Laws of Manu. Penguin Books, London
